# Heteronymous feedback from quadriceps onto soleus is influenced by limb loading and task context

**DOI:** 10.21203/rs.3.rs-5314064/v1

**Published:** 2024-11-25

**Authors:** Mark A. Lyle, Steven L. Wolf, Cristian Cuadra

**Affiliations:** Emory University School of Medicine; Emory University School of Medicine; University at Buffalo

**Keywords:** heteronymous reflex, proprioceptive feedback, task modulation, limb loading, femoral nerve

## Abstract

Heteronymous reflexes from quadriceps can increase and/or decrease soleus activity; yet few studies have examined factors influencing reflex strength. This study examined the independent influence of limb loading, posture, and task context on heteronymous feedback from quadriceps onto soleus. The influence of limb loading and posture was determined by comparing femoral nerve elicited heteronymous excitation and inhibition of soleus in a semi-recumbent position with and without 50% body weight limb loading and while standing with back support (n = 16). Task context was examined by comparing heteronymous reflex magnitudes while standing with back support to maintaining an unsupported squat posture which requires tonic soleus activity to maintain the posture (n = 12). Heteronymous inhibition decreased by 20% with limb loading in both semi-recumbent and standing postures, while excitation remained unchanged suggesting that limb loading, rather than postural orientation, independently modulates heteronymous inhibition. Inhibition decreased by 50% and excitation by 90% when maintaining the squat posture compared to supported standing, The pronounced suppression of both excitation and inhibition during the squat is considered a task-appropriate reflex modulation that aids in maintaining the posture. The results of this study highlight an important modulatory influence of limb loading afferents and task context on heteronymous reflex circuits.

## INTRODUCTION

Lower limb spinal reflexes contribute to motor coordination during locomotor tasks by influencing ongoing muscle activation^1–5^. The coordinating role of reflexes is achieved by task and context-dependent scaling of spinal reflex magnitudes such that their influence on muscle activation appropriately facilitates task goals. In humans, most research has focused on modulation of the soleus H-reflex, an electrical analog of the stretch reflex, that provides an estimate of the excitatory muscle spindle contribution to soleus activation and thus ankle plantarflexion. The soleus H-reflex is modulated across postures^6^ (e.g., supine to standing), phases of walking^7–12^, hopping^13,14^, and landing from a jump^15,16^ For example, the size of the H-reflex is small during the swing phase of walking when soleus activation would impair dorsiflexion, whereas the H-reflex increases during the stance phase when excitatory activation of soleus can contribute to ankle propulsion. Thus, characterizing the modulation of reflexes across tasks can help determine if the reflex normally functions during a task.

**Figure 1:**
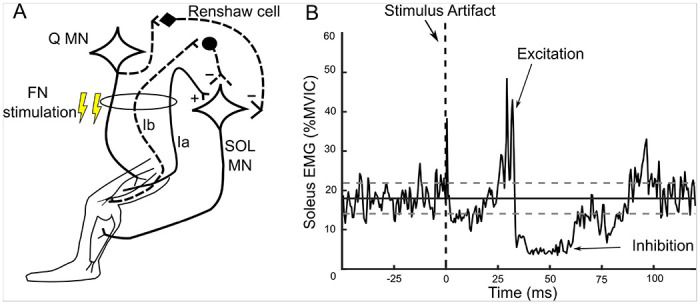
Schematic depicting the reflex circuits relevant for this study (A), and an example soleus EMG trace showing heteronymous excitation and inhibition in the semi-recumbent limb unloaded condition from a representative participant (B). A: Femoral nerve stimulation was used to evoke heteronymous excitatory Ia (continuous line), inhibitory Ib, and recurrent inhibition from antidromic motor axon propagation acting on Renshaw inhibitory interneurons (i.e., recurrent inhibition, dashed lines) from Q onto SOL motoneurons. B: Ensemble average of a rectified SOL EMG trace (mean of 30 repetitions) showing an excitatory period starting ~25 ms after FN stimulation followed by an inhibitory period starting ~32 ms. Excitation and inhibition onset were determined by the rectified trace moving above or below 1 standard deviation (horizontal dashed lines) of the mean SOL background EMG (grey horizontal line), respectively. Q, quadriceps; MN, motor neuron; FN, femoral nerve; SOL, soleus.

While task dependent modulation of H-reflex size has been extensively studied, few studies^6,17–23^ have examined modulation of heteronymous reflexes despite their potential to impact lower limb coordination. Heteronymous reflexes from a muscle can influence lower limb coordination by both increasing and decreasing motor output of other limb muscles. For example, excitatory length feedback (i.e., muscle spindles) and inhibitory force related feedback (i.e., Golgi tendon organs and recurrent inhibition) from the quadriceps muscles act to increase and decrease soleus motor output when sitting (See Fig. 1A). Thus, task-appropriate modulation of heteronymous reflexes may be even more important than that of the soleus H-reflex, because the magnitude of quadriceps excitation and inhibition of soleus could be in conflict depending on the motor task. Prior work suggests that, when compared to sitting, the magnitudes of heteronymous excitation and inhibition from quadriceps onto soleus decrease during standing^6,17^. Heteronymous inhibition from quadriceps onto soleus is also decreased when maintaining a squat posture compared to sitting^6^. However, the factors responsible for modulation of heteronymous feedback from quadriceps onto soleus remain unknown. The modulation of excitatory and inhibitory reflexes when standing compared to sitting could arise from peripheral factors such as limb loading^24,25^ and postural orientation^18,20^. As suggested by Barbeau et al. 2000, the reduction of heteronymous inhibition from quadriceps onto soleus when maintaining a squat posture could be further influenced by task context reflecting supraspinal descending control. Because tonic SOL activity is needed to maintain the squat posture, we consider task context dependent suppression of heteronymous inhibition as desirable to meet task goals and prevent postural instability. A better understanding of the factors that influence modulation of heteronymous reflexes is necessary to clarify the role of heteronymous reflexes in normal coordination and movement disorders.

The purpose of this study was to examine the differential influence of limb loading, posture, and task context on heteronymous excitation and inhibition from quadriceps onto soleus. To differentiate the influence of limb loading and posture on heteronymous reflexes, we evaluated heteronymous feedback from the quadriceps muscles onto ongoing soleus EMG in a supine position with and without 50% body weight loading, and while standing with wall support. Additionally, to test the modulatory influence of task-context, heteronymous excitation and inhibition was compared while standing with wall support to that during an unsupported squat position. Based on evidence that limb loading, and postural orientation can independently influence reflex strength^7,10,18,20,24^, we hypothesized that heteronymous excitation and inhibition from quadriceps onto SOL would decrease due to limb loading and would further decrease when in an upright posture (i.e., heteronymous excitation/inhibition during supine unloaded > supine loaded > standing with wall support). We further hypothesized that heteronymous excitation and inhibition from quadriceps onto soleus would decrease when maintaining a squat posture compared to supported standing due to the requirement of soleus to maintain postural stability.

## METHODS

Sixteen healthy participants (6 males and 10 females, 20 to 41 years) with no recent history of lower limb injury or neuromotor disorder that could effect participation were recruited for this study. Participants provided written informed consent in accordance with procedures approved by the Emory University Institutional Review Board with the ethical standards per the 1964 Declaration of Helsinki and its later amendment.

### Equipment

All data were acquired at 2000 Hz using an MP150 acquisition unit (Biopac Systems Inc, Goleta, CA, USA). Surface electromyography (EMG) was recorded from soleus (SOL) and vastus lateralis (VL) muscles with a pair of self-adhesive Ag-AgCl electrodes (2.2 × 3.5 cm; Vermed, Buffalo, NY, USA) and SignaGel (Parker Laboratories, INC. Fairfield, NJ). Prior to electrode placement, the skin was gently abraded and cleaned with isopropyl alcohol. SOL electrodes were positioned in the midline of the posterior aspect of the shank just distal to the gastrocnemius. The VL electrode pair was placed on the distal third of the muscle belly and aligned parallel to the direction of the muscle fibers. The reference electrode was placed at the proximal medial tibia. Surface EMG signals were differentially amplified, and hardware filtered (10-1000 Hz, AMT-8, Bortec Biomedical, Calgary, AB, Canada).

### Experimental Procedures

#### Soleus maximal voluntary isometric contraction (MVIC)

Maximal SOL EMG activity was recorded during voluntary isometric contractions with participants positioned semi-recumbent on a plinth with the hip in 20–30° of flexion, the knee fully extended, and the ankle secured in a neutral position with an ankle immobilizer boot (United Orthopedics, ROM Walker, Fort Wayne, IN, USA). Participants completed three maximal voluntary isometric contraction (MVIC) trials of 5 s duration. Verbal encouragement and SOL EMG feedback were provided to facilitate the best effort. The SOL EMG was bandpass filtered (10–500 Hz, zero-phase lag), rectified, and a 400 ms moving average was used to identify the peak SOL activity for each trial. The mean of the three trials determined maximal SOL EMG and was used to normalize EMG values to provide visual feedback during conditioning trials.

### Femoral nerve stimulation

The femoral nerve (FN) was stimulated (1 ms pulse width) with the cathode (3x2 cm electrode) positioned in the femoral triangle just medial to the sartorius and just distal to the inguinal ligament and the anode was positioned on the posterolateral buttock (7.5 x 13 cm, ValuTrode, Axelgaard Co., Ltd, CA, USA). The cathode was systematically moved to identify the location that produced a VL quadriceps M-wave with the lowest intensity. Compression of the cathode was provided by an elastic wrap secured around the waist and upper thigh. The maximal M-wave (Mmax) was identified by increasing the FN stimulation current by approximately 5 mA until the M-wave size no longer increased. Mmax was identified for each of the testing postures (semi-recumbent, standing, quarter squat).

### Evaluating heteronymous excitation and inhibition from quadriceps onto soleus EMG

Heteronymous excitation and inhibition from Q onto ongoing SOL EMG were examined by stimulating the FN while participants maintained SOL activity between 20 ± 3% MVIC. The tonic 20% SOL EMG was chosen based on prior literature demonstrating that heteronymous feedback can be observed as increase (excitatory) and decrease (inhibition) of this level of SOL activity^26–28^. Visual feedback of SOL EMG was provided, and FN stimulation was software triggered when participants maintained SOL EMG within the target EMG range for at least 3 seconds. The FN was stimulated at an intensity to produce 30% of Mmax in VL for all testing conditions. The peak-peak VL M-wave size was monitored, and the stimulation intensity adjusted to maintain VL M-wave size at 30% Mmax. Twenty-five to thirty-five repetitions were recorded during each of the task conditions with at least 10 seconds of rest between each repetition.

The modulatory influence of limb loading and posture on heteronymous excitation and inhibition from Q onto ongoing SOL EMG was examined by testing participants 1) while lying on a plinth in a semi-recumbent posture, 2) while lying on a plinth in a semi-recumbent posture with the test limb loaded to 50% body weight, and 3) while standing with their back supported against a wall (i.e., 50% body weight on test limb). During each of the three conditions, the participants’ posture was 20–30° of hip flexion, full knee extension, and the ankle was in a neutral position. In the semi-recumbent positions, isometric ankle plantarflexion to produce the tonic SOL EMG target of 20% MVIC was achieved by wearing an ankle immobilizer boot. To achieve limb loading of 50% body weight in the semi-recumbent posture, the plantar surface of the ankle immobilizer boot was positioned against a motorcycle scissor jack (Vivohome, Ontario CA) that was in series with a force plate (MU1117, Bertec, Columbus, OH) fixed to the wall. While an experimenter applied pressure to the shoulders to prevent superior body translation, the scissor jack was slowly advanced to load the limb until 50% body weight was achieved by monitoring the real-time force. In a subset of 14 of 16 participants, the independent influence of the ankle immobilizer boot on heteronymous feedback from Q onto SOL was recorded when the limb was loaded without the ankle immobilizer boot.

In 12 of 16 participants, the effect of task context was examined by recording heteronymous feedback from Q onto SOL while participants stood in an unsupported quarter squat posture with the hip approximately 30–45 degrees, knee flexed ~ 40 degrees, and ankle slightly dorsiflexed. The participants would hold the squat posture, typically for 3–8 seconds, until the background SOL EMG triggered the FN stimulation. Participants would return to upright standing between repetitions.

### Data Analysis

All data were processed off-line using routines written in MATLAB R2021a (MathWorks, Natick, MA). The peak-to-peak M-wave in VL was calculated during all testing conditions after bandpass filtering (0.5–500 Hz, zero phase shift) to ensure the population of axons activated by FN stimulation was consistent. Heteronymous excitatory and inhibitory effects from FN stimulation onto SOL EMG were evaluated after the EMG was bandpass filtered (10–500 Hz, zero phase shift) and rectified. The rectified SOL EMG was normalized to each participant’s MVIC value and averaged across the 25–35 repetitions. The background SOL EMG means and standard deviations (SD) were calculated over a 400 ms period before stimulation.

The pre-stimulus background SOL EMG mean and SD were used to identify excitation and inhibition onset, duration, and magnitudes as described in detail previously (Fig. 1B)^26,27^. Excitation onset was determined as the time point when the SOL EMG exceeded 1SD above the mean for ≥ 2 ms. The end of excitation was determined as the time at which the SOL EMG trace returned below the 1 SD line for a period of ≥2 ms. Only excitatory responses with onset ≥ 23 ms were considered as arising from heteronymous Ia facilitation^29,30^. Inhibition onset and termination were determined as the SOL EMG moving 1SD below the mean background SOL EMG for a period of ≥2 ms and returning above the 1 SD line for ≥ 2 ms. Inhibitory responses were considered only if the onset was < 45 ms since the fastest transcortical effects could manifest soon thereafter. The duration of excitation and inhibition were calculated as the difference between effect onset and termination. The excitation and inhibition magnitudes were calculated as the area relative to background SOL EMG using trapezoidal numerical integration (trapz in Matlab, SOL %MVIC× ms). Heteronymous excitation magnitudes are reported as positive values, whereas heteronymous inhibition is expressed as negative values.

### Statistical analysis

All statistical analyses were performed with R-Studio, version 1.3.1073 (© 2009–2020 RStudio, PBC). Descriptive statistics are reported in the text as mean ± standard deviation. Quantile-quartile plots and Shapiro-Wilks test were used to assess normality for each variable and condition separately. To test the effect of limb loading and posture on heteronymous feedback from Q onto SOL (n = 16), repeated measures analyses were conducted using separate linear mixed effects models for heteronymous excitation and inhibition with task as a fixed factor (3 levels: semi-recumbent unloaded, semi-recumbent with limb loaded 50% body weight, and standing with wall support). The *F*-values were computed using a compound symmetry variance-covariance structure and the Kenward-Roger method. Pairwise comparisons were completed with Bonferroni correction. The SOL pre-stimulation background EMG and peak-to-peak M-wave size were examined with linear mixed models as described above across the three task conditions.

The influence of task context was determined by comparing the magnitudes of heteronymous excitation and inhibition from Q onto ongoing SOL EMG during supported standing with participants’ back leaning against the wall and when maintaining a quarter squat posture with paired t-tests or Wilcoxon signed rank tests (n = 12). The SOL pre-stimulation background EMG and peak-to-peak M-wave size were examined with paired t-tests.

To determine the influence of the ankle immobilizer boot on heteronymous feedback, a paired t-test was used to compare heteronymous excitation and inhibition from Q onto SOL during the semi-recumbent limb loaded condition with and without the immobilizer boot.

## Results

### Effect of limb loading and posture on heteronymous reflex feedback from Q onto SOL

**Figure 2:**
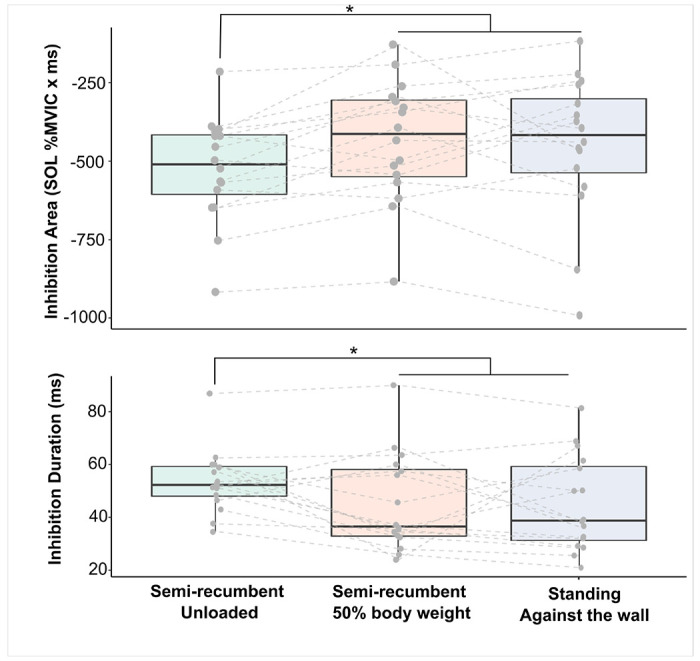
Comparison of heteronymous inhibition area (top) and duration (bottom) when semi-recumbent with the limb unloaded, semi-recumbent with the limb loaded 50% of body weight, and standing with wall support. A significant reduction of inhibition area (*P*= 0.01) and duration (*P*= 0.042) was observed in the semi-recumbent limb loaded and standing posture compared to the semi-recumbent limb unloaded condition.

Heteronymous inhibition was observed in all participants in the recumbent limb unloaded and loaded conditions, and when standing with back support (Fig. 2). There was a significant effect of task condition on the magnitude of heteronymous inhibition measured as a decrease in SOL EMG area (*F*_2,30_ = 5.37, *P* = 0.01) and the duration of inhibition (F_2,30_ = 3.53, P = 0.042). Post-hoc analysis showed greater inhibition when the limb was unloaded (−525.68 ± 166.61%MVIC × ms) when compared to the semi-recumbent position with limb loaded to 50% body weight (−434.67 ± 192.83%MVIC × ms, *P* = 0.007) and when standing with back against the wall (−449.41 ± 227.49%MVIC × ms, *P* = 0.018). No difference was found for inhibition area between the semi-recumbent limb loaded and the supported standing position (*P* = 0.62). A similar trend was found for inhibition duration where a longer duration was observed when the limb was unloaded (53.50 ± 11.96 ms) compared to when the limb was loaded in the semi-recumbent position (45.3 ± 18.38 ms, *P* = 0.0317) and when standing against the wall (45 ± 17.99 ms, *P* = 0.026). No difference was found between the inhibition duration between the semi-recumbent limb loaded and the supported standing position (*P* = 0.93). The onset of heteronymous inhibition was not different between task conditions (*F*_2,30_ = 0.55, *P* = 0.58, 36.3 ± 2.38 ms).

Heteronymous excitation was observed in 12, 13, and 14 of the 16 participants across the semi-recumbent unloaded, semi-recumbent limb loaded, and supported standing with back against the wall conditions, respectively. However, heteronymous excitation magnitudes from Q onto SOL as measured by SOL EMG area was not influenced by changes in limb loading or the upright posture (*F*_2,23.74_ = 0.47, *P* = 0.629, 92.9 ± 49.6, 113.9 ± 72.3, and 106.6 ± 85.6%MVIC x ms). In addition, there was no difference across task conditions for heteronymous excitation duration (*F*_2,23.85_ = 1.17, *P* = 0.327, 5.179 ± 1.575 ms) nor the excitatory onset latency (*F*_2,23.24_ = 1.84, *P* = 0.182, 30.244 ± 2.471 ms).

Experimental control of pre-stimulus SOL background EMG and FN stimulation-evoked M-wave amplitudes were achieved across the task conditions. Participants consistently attained the SOL EMG target range across the semi-recumbent unloaded, semi-recumbent 50% body weight loaded, and standing with back supported against the wall (*F*_2,30_ = 1.94, *P* = 0.162, 18.0 ± 1.43%MVIC × ms). The FN stimulation evoked M-wave amplitude was not different across task conditions (*F*_2, 28.1_ = 0.59, *P* = 0.56, 30.2 ± 2.42% MMax) suggesting that a consistent population of sensory and motor axons were recruited.

A paired t-test was used to examine the possibility that wearing the ankle immobilizer boot influenced the magnitudes of heteronymous inhibition and excitation since the boot required firm compression against the shank. There was no significant difference in the magnitudes of heteronymous inhibition *t*(_13,0.05_) = −1.335, P-value = 0.2047, overall mean= −456.012 ± 173.51) or excitation (*t*(_10,0.05_) = 0.571, P-value = 0.5806, overall mean = 110.945 ± 70.953%MVIC × ms) from Q onto SOL during the semi-recumbent limb loaded condition when completed with or without the immobilizer boot indicating there was no effect of shank compression on heteronymous feedback.

### Effect of task context on heteronymous reflexes from Q onto SOL

**Figure 3:**
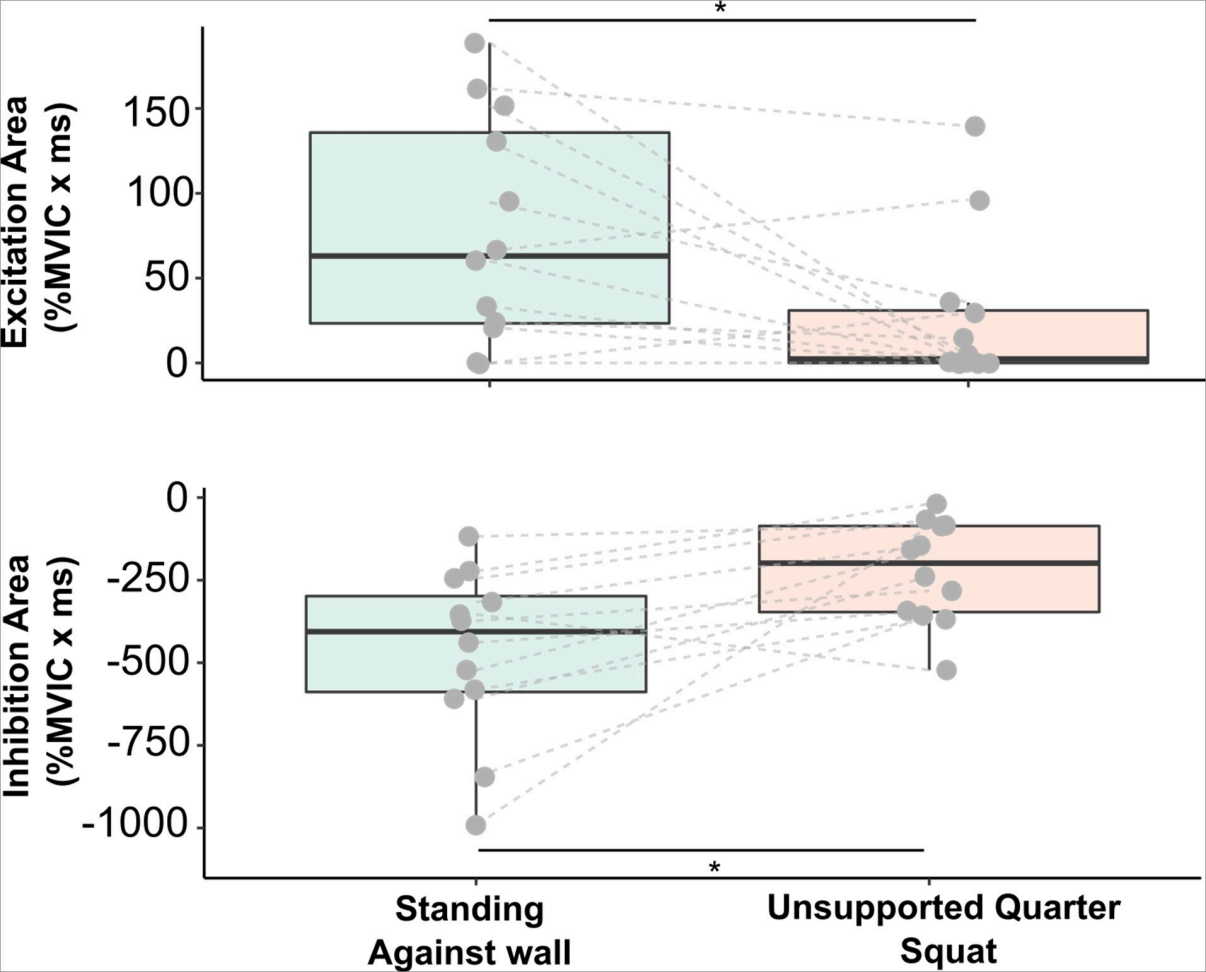
Heteronymous excitation (top panel) and heteronymous inhibition (bottom panel) magnitudes when standing with back support against the wall and when maintaining a squat posture. Heteronymous excitation (*P=0.011) and inhibition (*P= 0.009) were significantly decreased when maintaining the squat posture compared to standing with back against the wall.

The effect of task context was assessed in 12 participants by comparing heteronymous feedback from Q onto SOL when participants stood supported with their back against the wall with that during an unsupported quarter squat position (Fig. 3). Whereas there is minimal consequence of heteronymous inhibition and excitation from Q onto SOL when standing with back against the wall, heteronymous inhibition of SOL is undesirable during the squat condition because tonic soleus activity is required to maintain the posture (i.e., task context). Heteronymous excitation was observed in 12 of 12 participants during supported standing (Fig. 3, top panel). Heteronymous excitation was significantly decreased when maintaining the squat posture compared to standing with back against the wall (Wilcoxon = 151, *P* = 0.011, *median ± quartiles* 63 ± [ 23.3, 13.7] %MVIC × ms and 2.35 ± [ 0, 30.971] %MVIC × ms, respectively).

Heteronymous inhibition as measured by SOL EMG area was significantly decreased during the unsupported quarter squat compared to supported standing with back against the wall (−222.68 ± 153.98 vs. −468.03 ± 257.86%MVIC × ms, *t*(_11,0.05_) = −3.15, *P* = 0.009, Fig. 3, bottom panel). In addition, inhibition duration was significantly shorter (*t*(_11,0.05_) = 4.62, *P* = 0.0007) for the unsupported quarter squat compared to supported standing (21.38 ± 9.59 vs. 44.5 ± 17.96 ms). No difference was observed between tasks for inhibition onset (*t*(_11,0.05_) = −1.58, *P* = 0.144, 36.8 ± 2.67 ms).

Experimental control of pre-stimulus SOL background EMG and FN stimulation evoked M-wave amplitudes were achieved during the supported standing and squat postures. Participants’ pre-stimululation background SOL EMG was not different when standing with the back leaning against the wall compared to maintaining the unsupported squat posture (*t*_11,0.05_ = 1.368, *P* = 0.196, 18.5 ± 2.51%MVIC × ms). In addition, the FN stimulation evoked M-wave amplitudes were not different between task conditions (*t*_11,0.05_ = 0.155, *P* = 0.879, 27.7 ± 3.39% MMax) indicating a consistent population of sensory and motor axons were recruited.

## DISCUSSION

The purpose of this study was to evaluate the differential influence of limb loading, posture, and task context on heteronymous reflexes from quadriceps onto soleus. We found that, compared to a supine limb unloaded condition, heteronymous inhibition decreased when the limb was bearing half body weight, whether positioned in a supine or in an upright supported standing posture. These findings indicate an independent modulatory effect from limb loading but not postural orientation. In addition, we found that when compared to a supported standing posture, participants’ heteronymous inhibition and excitation decreased while maintaining an unsupported quarter squat posture. These findings support a modulatory influence of task context on heteronymous reflexes, because ongoing soleus activation is needed to maintain the squat posture and thus inhibition would have negative task consequences. Together, these findings indicate limb loading and task context have a significant modulatory influence on heteronymous reflexes from quadriceps onto soleus.

Numerous studies have shown that FN stimulation elicits heteronymous inhibition and excitation onto SOL when in a seated or semi-recumbent posture^6,26,27,29–32^. The heteronymous inhibition has been attributed to force related feedback (i.e., Golgi tendon organs, recurrent inhibition)^26–28,32^ and can be substantial, reducing tonic SOL EMG held at 20% of maximum by half or more and lasting greater than 50 ms. Heteronymous excitation arises from length related muscle spindle Ia afferents^31^. A strong inhibition and/or excitation of SOL motor output by Q could be undesirable depending on the task and circumstance, yet few studies have examined modulation of heteronymous feedback from Q onto SOL.

### Heteronymous inhibition is modulated by limb loading

Identifying factors responsible for reflex modulation across different postures and tasks provides important insight regarding the neural control and function of reflex circuits. Prior work has shown that limb loading^10,33^ and head-body orientation relative to gravity^34–36^ can independently modulate soleus H-reflex strength. The current study was designed to examine the independent influence of limb loading afferents and postural orientation encoded by the vestibular system on heteronomous inhibition and excitation from Q onto SOL for the first time. This goal was achieved by comparing heteronymous spinal reflex feedback from Q onto SOL during three postural conditions: semi-recumbent with the limb unloaded, semi-recumbent with the limb loaded to half body weight, and standing with back supported against a wall such that each limb was loaded with half body weight. The standing posture involved leaning with participants’ back against the wall to eliminate the need for active body control; thus, the principal difference between the semi-recumbent limb loaded and standing conditions was postural orientation.

The magnitude and duration of heteronymous inhibition and excitation in the supine unloaded condition in this study is similar to a prior report when in a more upright seated posture^26^. Whereas heteronymous excitation was the same across the semi-recumbent and supported standing task conditions, heteronymous inhibition was decreased by 20% when the limb was loaded both in supine and in the wall supported standing posture when compared to the semi-recumbent unloaded condition. These data indicate afferents arising from limb loading had a suppressive influence on heteronymous inhibition. While this study was not designed to identify the specific afferents, prior work suggests cutaneous and joint afferents are plausible candidates^24,37^. In contrast to our hypothesis, our results indicate a change in tonic vestibulospinal input accompanying the upright postural orientation did not exert an independent modulatory effect on heteronymous inhibition. We expected heteronymous inhibition would be further suppressed when standing compared to the semi-recumbent loaded condition based on prior studies demonstrating an independent influence of postural orientation on other reflexes^34,36^. The lack of an additional suppression of heteronymous inhibition during supported standing in this study may be due to the limited task consequences of inhibition since the posture was stable with back support and the feet were positioned anterior to body center of mass.

### Heteronymous inhibition and excitation from Q onto SOL are modulated by task context

An additional important finding of this study was that task context had a significant modulatory influence on heteronymous feedback from Q onto SOL. When compared to the supported standing condition, heteronymous inhibition decreased by about 50% and excitation by almost 90% when maintaining a quarter squat posture. This finding is consistent with Barbeau, et al. ^6^, who found heteronymous inhibition decreased by approximately 40% when maintaining a squat posture compared to sitting. Our results indicate that a similar magnitude of modulation occurs between standing tasks, and thus the findings from the present study highlight that the modulation is independent of limb loading. Importantly, the Q and SOL muscles work synergistically to maintain the quarter squat posture making heteronymous inhibition and excitation of SOL destabilizing. Thus, we concur with Barbeau, et al. ^6^ that heteronymous inhibition and excitation can be strongly modulated based on task context, where functional consequences of increased inhibition and excitation could be undesirable, and lead to poor performance of the task. In this study, reduced heteronymous inhibition and excitation is interpreted as a desirable change in reflex strength that supports the task goal of maintaining the squat posture. Additional studies are warranted to examine modulation of heteronymous feedback during additional tasks in persons with and without neurological impairments. The consensus is that context dependent modulation of reflexes arises from descending supraspinal modulation^38^ raising the possibility that reflex modulation could be impaired in persons with damage to supraspinal centers such as post-stroke. Further study of task-context dependent modulation of heteronymous reflexes will help clarify their functional role and potential contribution to movement impairments.

### Methodological considerations:

The study was designed to evaluate the independent influence of limb loading, postural orientation, and task context. Several procedural considerations and alternative interpretations must be considered. A primary finding of this study was that heteronymous inhibition was decreased by limb loading but postural orientation did not have an independent contribution. This interpretation was based on heteronymous inhibition magnitudes being the same when the limb was loaded to half body weight when semi-recumbent and standing. However, we did not evaluate heteronymous inhibition when upright without limb loading. Therefore, while the upright posture did not contribute an additional modulation of heteronymous inhibition, further work examining heteronymous feedback using more head-trunk orientations relative to gravity with and without limb loading would more directly identify the independent effect of tonic vestibular actions on heteronymous feedback.

Reduced heteronymous feedback from Q onto SOL when maintaining the squat posture compared to back supported standing was interpreted as purposeful context-dependent neural modulation, likely arising from supraspinal centers^6,38^. However, the squat posture requires additional muscles to provide stabilization. While soleus muscle activation was the same for the supported standing and squat posture, other muscle activation magnitudes and head-trunk orientation were not matched across conditions. Thus, in addition to task context, we cannot rule out a modulatory influence from peripheral factors other than limb loading, such as trunk activation or head-trunk orientation.

When examining heteronymous feedback in the semi-recumbent posture, an ankle immobilizer boot was used to secure the ankle position so participants could complete isometric, tonic SOL EMG to 20% of maximal. To evaluate whether compression of the shank from the boot influences heteronymous feedback, we compared heteronymous inhibition and excitation between the supine limb loaded condition with and without wearing the ankle immobilizer in a subset of participants. The lack of differences in heteronymous feedback between conditions suggest the limb loading afferents were responsible for the decreased heteronymous inhibition without an additional influence from compression of the shank by the immobilizer boot.

## Conclusion

This study examined the differential influence of limb loading, postural orientation, and task context on heteronymous excitatory and inhibitory reflexes from Q onto SOL. We found that limb loading, but not postural orientation, resulted in a significant decrease in heteronymous inhibition. We further observed a marked decrease in heteronymous inhibition and excitation from Q onto SOL when the heteronymous feedback had the potential to negatively influence task performance (i.e., maintain a quarter squat posture). Together, the findings from this study indicate an independent modulatory influence from limb loading afferents and task context on heteronymous reflex circuits from Q onto SOL.

## Data Availability

All data supporting the results in the manuscript are shown in the figures. The data are available from the corresponding author on reasonable request.
